# The Epigenomics of Embryonic Pathway Signaling in Colorectal Cancer

**DOI:** 10.3389/fphar.2017.00267

**Published:** 2017-05-19

**Authors:** Curt Balch, Jayaram B. Ramapuram, Amit K. Tiwari

**Affiliations:** ^1^Department of Pharmacology and Experimental Therapeutics, School of Pharmacy, University of Toledo, ToledoOH, USA; ^2^Bioscience Advising, YpsilantiMI, USA; ^3^Complex Biological Systems Alliance, North AndoverMA, USA; ^4^Department of Drug Discovery and Development, Auburn University, AuburnAL, USA

**Keywords:** colorectal cancer, embryonic signaling pathways, epigenomics, epithelial-to-mesenchymal transition, tumor progression

## Abstract

Colorectal cancer (CRC) is the second-leading cause of cancer death in developed countries. While early detection (e.g., colonoscopy) generally yields excellent outcomes, metastatic and drug-resistant disease is uniformly fatal, and non-compliance for screening remains over 25%. Familial CRCs (10% of total cases) primarily include mutations in the gene *APC*. Somatic disease is linked to several environmental several risk factors, including mutations in *WNT, KRAS*, and *TGF*β. To reflect the genesis/progression of CRC, a series of five discrete stages, from normal colon mucosa to fully invasive carcinoma, each regulated by specific “gatekeeper” genes, remains well-accepted after 20 years. However, many CRC tumors do not possess those particular mutations, suggesting alternative mechanisms. More recently, embryo-like “cancer stem cells” have been proposed to undergo self-renewal and drive tumorigenesis (and possibly, metastasis), as governed by specific “epigenomic” alterations. Here, we review recent literature describing possible mechanisms that underlie these phenotypes, including cancer “stemness,” believed by many to associate with the epithelial-to-mesenchymal transition (EMT). We further propose that the maintenance of undifferentiated phenotypes, by the activity of distinct transcription factors, facilitates chromatin remodeling and phenotypic plasticity. With that regard, we support recent assertions that EMT is not an “either/or” event, but rather a continuous spectrum of mesenchymal vs. epithelial phenotypes (in various degrees of aberrant differentiation/undifferentiation). Finally, we discuss possible methods of pharmacologically targeting such aberrant epigenomes, with regard to their possible relevance toward halting, or even reversing, colorectal cancer progression.

## Introduction

Colorectal cancer (CRC) is the second-leading cause of cancer deaths in the United States, with an estimated 50,130 deaths in 2014, a national expenditure of $14 billion, and an individual lifetime risk of 1 in 20. While the 5-year survival rate for localized CRC is >90%, only 40% of cases are detected at this (largely asymptomatic) stage, and for metastatic disease, survival falls to 8–12%. The risk of CRC increases with age (>50 years), diets high in red and processed meats, sedentary lifestyle, obesity, history of inflammatory bowel disease, and smoking. Non-compliance with screening for these at-risk individuals remains approximately 25% (Wong et al., 2016), and 5–10% of CRC cases are due to hereditary conditions, the majority of which include mutations in the tumor suppressor gene *APC*. Consequently, while optimism persists for increased compliance with preventative screening, possibly due to healthcare reforms to alleviate cost, it appears a large number of advanced cases will remain (Siegel et al., 2014).

In this review, we set forth a model whereby “epigenomic” anomalies in normal colon crypt stem cells, and their terminally differentiated progeny cells, transform to an embryonic-like state, followed by further benign and malignant progression. Consideration of these mechanisms may support further study of “epigenetic” therapies for this life-threatening pathology.

## Initiation and Pathology of Colorectal Cancer

### Colon Development

The colon is a quadrate-shaped organ possessing six substructures, facilitating progression of solid waste through the cecum (attached to the ilium of the small intestine), which is then transported through the ascending, traverse, descending, and sigmoid colon, which attaches to the rectum/anus (Colon Cancer Alliance, 1983) The colon serves to remove water, salt, and nutrients throughout the progressive elimination of solid waste, in symbiosis with over 700 species of bacteria and various simple eukaryotes (gut flora) (Colon Cancer Alliance, 1983). Six major colonic cell types include surface columnar epithelial (absorptive “colonocytes”), goblet (mucus-secreting) cells, vacuolated cells, deep crypt secretory cells, M-fold cells, and crypt colonic stem cells (Colony, 1996).

### Colorectal Tumorigenesis

Colorectal cancer is well-accepted to proceed through five distinct stages: (1) aberrant foci; (2) small adenoma (adenomatous polyps); (3) large adenoma; (4) adenocarcinoma; and (5) invasion/metastasis. The pathology of non-familial CRCs is often associated with overactivity of the epidermal growth factor receptor (EGFR), mutations in *Wnt*, loss of *APC*, activating *KRAS* mutations, and mutations in *NRAS*, *BRAF* (8–12%), *PIK3CA*, and *TP53* (50% of cases). Additionally, a balance between “gatekeeper” (anti-proliferation), “caretaker” (maintain genomic stability) and “drivers” (oncogenes) has been hypothesized to regulate other CRC phenotypes, including (1) the development of microsatellite instability (MSI) (due largely to loss of DNA mismatch repair), leading to overactivity of COX-2, EGFR, and/or Wnt pathways (leading to small adenomas); (2) *KRAS* and/or *PIK3CA* pathway overactivity (leading to large adenomas); and (3) inactivation of the tumor suppressor gene *TP53* and downregulation of TGF-β signaling, leading to invasive/metastatic carcinomas (Wang et al., 2002; Markowitz and Bertagnolli, 2009; Vogelstein and Kinzler, 2015). With regard to specific gene mutations in non-hereditary (i.e., somatic) CRC, *APC* is mutated in 85%, *TP53* in 40–50%, *PIK3CA* in 35% (activating mutations), and *TGFBR2* in 45–50% of sporadic CRCs (Markowitz and Bertagnolli, 2009).

While there is hope that increased screening (e.g., colonoscopy, laproscopy, highly sensitive human occult fecal blood testing, etc. for persons over age 50) compliance will further increase early detection and treatment, due to increased education, access to health insurance, etc. However, at present, the only option for unresectable metastatic disease is conventional cytotoxic chemotherapy. While response to those initial chemotherapy regimens usually occur, resistance generally ensues within 6 months. Life-extending agents include conventional cytotoxics, such as 5-fluoracil, capecitabine, and topotecan, in various combinations with one another, and “targeted” therapeutics, such as bevacizumab (VEGFR antagonist) and the EGFR-inhibitory drugs cetuximab and panitumumab (Aparo and Goel, 2012; Recondo et al., 2014), Unfortunately, resistance to these agents eventually ensues, with a mere 13.3-month subsequent survival rate (O’Connell et al., 2008).

### Epigenetics

Although mutated genes unequivocally contribute to the progression of CRC, gatekeeper, caretaker, and driver genes are not always altered in all CRC stages (Feinberg et al., 2006). Consequently, many cancer investigators are now focusing on the importance of epigenetics to the phenotypic plasticity that is crucial for CRC tumor progression (Tam and Weinberg, 2013; Pereira et al., 2015). Epigenetics refers to the regulation of gene transcription absent DNA-coding sequence alterations, and includes post-translational modifications to DNA and histones, nucleosome repositioning, and microRNA regulation of mRNA translation and/or stability (Goel and Boland, 2012). The most well-known of these, methylation of deoxycytosine (commonly referred to as the “fifth base” comprising DNA), within CG dinucleotides, is universally throughout higher eukaryotes, particularly in heterochromatin and large intergenic regions (Beyersmann, 2000). However, specific CG-rich (>60%) regions of <1000 base pairs (“CpG islands”), associated with over 70% of gene promoters are protected from transcriptionally repressive DNA methylation in normal cells (Deaton and Bird, 2011). A hallmark of cancer is a redistribution of DNA methylation patterns, i.e., hypermethylation of CpG islands, often resulting in repression of tumor suppressor genes, and loss of methylation in heterochromatin, resulting in genomic instability (Rodriguez-Paredes and Esteller, 2011). Moreover, early deoxycytosine methylation may occur in DNA repair genes (e.g., MLH1, MSH2, etc.), further favoring MSI and genetic mutations (Feinberg et al., 2006).

### Epigenetics in Colorectal Cancer Development

In CRC specifically, it has been asserted that epigenetic changes occur very early in tumorigenesis. Specifically, MSI, and DNA methylation/silencing of DNA mismatch repair (MMR) genes, lead to a poorly understood phenomenon known as “CIMP” (***C***pG ***i***sland ***m***ethylator ***p***henotype). The CIMP phenotype involves widespread (but not random) promoter methylation throughout the genome (Issa, 2004). Moreover, it has been reported that typical metastatic CRCs possess ∼61 infrequently mutated genes, with 15 of these designated as driver genes, and the remainder representing mutated passenger genes (Markowitz and Bertagnolli, 2009). By contrast, one recent genome-wide analysis revealed that approximately 5% of the total CRC tumor genome (>1000 genes) harbors abnormal DNA methylation (Schuebel et al., 2007). Thus, it would appear prudent to consider both *genetic* and *epigenetic* aberrations in the etiology of CRC (You and Jones, 2012). For example, while 85% of sporadic CRCs possess transcriptional loss of *APC*, in addition to inactivating mutations, this gatekeeper can also be silenced by promoter DNA methylation (Lee et al., 2009).

Another example of epigenetic/genetic “crosstalk” during CRC tumorigenesis is the finding of genetic mutations in genes encoding epigenetic modifiers. For example, in a study of the genetic landscape of CRC, it was found that 50% of mutation-driver genes were those encoding epigenetic modifiers (e.g., *DNMT3b*, *EZH2*, etc.) (Vogelstein et al., 2013). Another genome-wide association study revealed mutations in the gene encoding the Tet methylcytosine dioxygenase 2, an enzyme involved in DNA demethylation (Toth et al., 2017). These (and many other studies) demonstrate the complex interplay between genetics and epigenetics (Feinberg et al., 2006), thus increasing the challenges of designing targeted CRC therapies.

Epigenetics likely also contributes to the wide degree of heterogeneity of CRC tumors. Recently, an international consortium recognized four CRC consensus molecular subtypes, including: (1) CMS1 (microsatellite instable, hypermutated); (2) CMS2 (activated canonical WNT and MYC signaling; (3) CMS3, with metabolic dysregulation; and (4) CMS4, having activated TGF-β signaling, with stromal invasion and angiogenesis. Each of these subtypes was found to have their own distinctive epigenomes (Guinney et al., 2015). Moreover, CRC tumors with relative epigenomic homogeneity associated with short relapse-free and overall survival (Martinez-Cardus et al., 2016).

### Epithelial-to-Mesenchymal Transition

The importance of epigenomics to cancer is further supported by studies in melanoma, in which plating of melanoma cells onto an extracellular matrix derived from embryonic stem cells, could reverse the malignant phenotype (Costa et al., 2009). Such malignant reversion was later found to associate with epigenetic remodeling by microenvironmental paracrine release of the cytokine Lefty, which is silenced by DNA methylation, and also by the microRNA miRNA-302, in melanoma cells. Analogously, Lefty is inhibited by the oncoprotein Nodal, and the embryonic transcription factor Notch4 (Costa et al., 2009; Barroso-delJesus et al., 2011). Other studies showed that the epithelial-to-mesenchymal transition (EMT)-opposing microRNAs miR-34 and the miR-200 family are silenced by DNA methylation in CRC metastatic cells and tissues. Re-expression of these miRNA genes, by DNA methylation inhibitors, inhibited the synthesis of the EMT modulators TWIST, SNAIL, and ZEB1 (Siemens et al., 2011; Roy et al., 2012; Hur et al., 2013). Moreover, a p53/miR-34 axis has been reported to stabilize the β-catenin antagonist GSK-3β within the nucleus, resulting in repression of Wnt and Snail pathway transcriptional targets (Kim et al., 2013).

**FIGURE 1:**
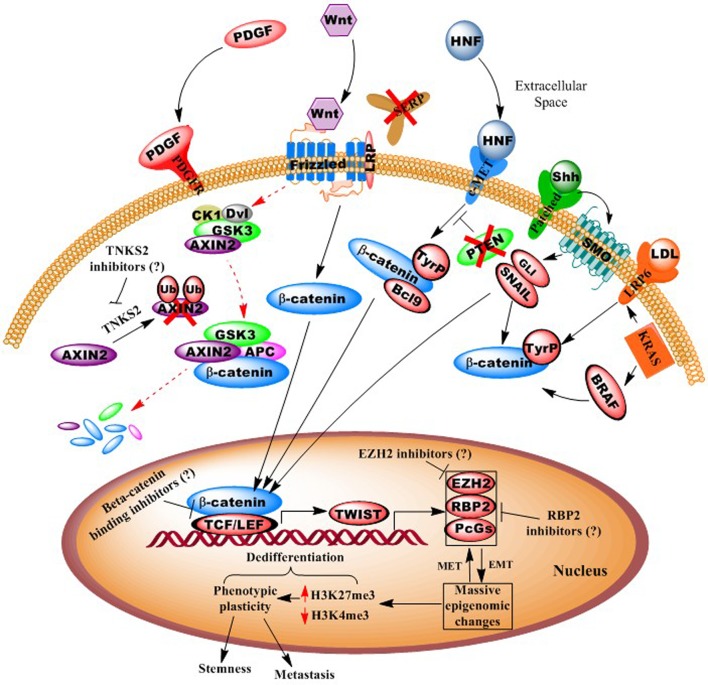
**Illustration of autocrine and paracrine signaling pathways in colorectal cancer**. Diagram shows how numerous oncogenic signaling pathways, including Wnt, PDGF, c-MET, and KRAS, converge to effect epigenomes that facilitate pro-neoplastic (“dedifferentiation”) gene transcription. Possible points of epigenetic interventions, potentially reversing dedifferentiating epigenomes, are shown. For example activation of epithelial-to-mesenchymal transcription factors (e.g., Twist) leads to whole-genome chromatin remodeling and phenotypic plasticity, which can be inhibited or reversed.

Consequently, we posit that an additional aspect of CRC neoplastic epigenomic alterations is their intimate role in regulating EMT (Tam and Weinberg, 2013), which has further been linked to the cancer “stemness” phenotype (Polyak and Weinberg, 2009). The EMT transition involves massive, genome-wide epigenomic changes that underlie phenotypic plasticity, such as the loss of tight junctions, loss of cell-to-cell adherence, loss of cell polarity, changes in cell morphology to an elongated shape (allowing traverse between endothelial cells), and the formation of lamellapodia and cytoskeletal remodeling, thus facilitating motility and invasion (**Figure 1**) (Hugo et al., 2007).

### Epigenomic Reactivation of Embryonic Developmental Pathways

In addition to EMT, most cancers are now known to reactivate embryonic self-renewal pathways, including *Hedgehog*, *Notch*, and *TGF*β/*Stat3*. As described above, most CRCs are reliant on the *Wnt* (*Wingless*) developmental pathway. It is possible that direct targeting of embryonic pathways might be more effective against both stem and dedifferentiating tumor cells (Takebe et al., 2011; Medema, 2013; Pattabiraman and Weinberg, 2014), and cancers “addicted to” upregulated embryonic pathway activity (e.g., Wnt in CRC and PIK3CA in metastatic breast cancer), combined with high tumor heterogeneity, might be more vulnerable to such therapies (Bienz and Clevers, 2000; Segditsas and Tomlinson, 2006; Hernandez-Aya and Gonzalez-Angulo, 2011). Another “master regulator” of chromatin remodeling to an undifferentiated phenotype is the Polycomb oncoprotein EZH2, which represses transcription by trimethylation of histone H3, lysine 27 (H3K27me3) (Chang and Hung, 2012).

## Current and Potential CRC Therapeutic Approaches

In **Figure 1**, we present a model for the genesis and progression of CRC, via canonical or non-canonical Wnt signaling. Here, the simplest means of carcinogenesis would be genetic or epigenetic anomalies in colonic crypt stem cells, particularly those expressing the Wnt signaling component LGR5 (Zeki et al., 2011; Carmon et al., 2012). However, epigenomic alterations also allow for dedifferentiation of villi cells (transient-amplifying or even fully differentiated, polarized colonocytes), particularly in response to inflammation (and activity of the oncogenic pathway NF-κB (Schwitalla et al., 2013), and various environmental agents (Haggar and Boushey, 2009). As >95% of CRCs are believed to involve overactive Wnt signaling, this may occur via numerous mechanisms, including *APC* mutation, DNA methylation silencing of *SFRP* (secreted frizzled receptor protein), activating mutations in beta-catenin, loss of AXIN2 degradation by silencing of the E3 ligase gene tankyrase (*TNKS2*), and upregulated signaling of the hepatocyte growth factor (HGF) pathway (Suzuki et al., 2004; Fodde and Brabletz, 2007; Schneikert and Behrens, 2007; Schwitalla et al., 2013).

In this scenario, reactivation of embryonic signaling pathways (possibly via aberrant paracrine interactions with a pericryptal myofibroblast “stem cell niche”) (Vermeulen et al., 2010) and CRC upregulation of Wnt signaling, in conjunction with the loss of gatekeeper genes (*SFRP*, *APC*, *DKK*, etc.) upregulate EMT and other promalignant processes (Aguilera et al., 2006; Silva et al., 2014). Additional activating mutations in CRC driver genes/pathways may also “crosstalk” (e.g., HGF, PI3K/AKT, TNKS2) with Wnt signaling to facilitate rapid proliferation into a fully malignant tumor (via β-catenin stabilization, nuclear translocation, and cotransactivation of TEF/LEF-occupied gene promoters). These events then initiate a cascade that eventually results in EMT and a cancer stem-like phenotype, involving highly plastic phenotypes, such as cell shape distortion (extravasation between endothelial cells), motility to reach the circulatory system, and suppression of immunosurveillence by downregulating specific HLA, and other antigenic cell surface immunophenotypes (Yaguchi et al., 2011).

## Possible Epigenome-Targeting Therapeutic Approaches for CRC

### Wnt Inhibitors

As mentioned above, *APC* and/or *CTNNB1* (β-catenin) mutation(s) are present in >90% of CRC cases, thus singling out the Wnt pathway as predominant in this cancer type (Ayadi et al., 2015). Moreover, Wnt ligands can stimulate various non-canonical and non-frizzled related pathways, and Wnt signaling has been found to “crosstalk” with several other signal cascades (e.g., KRAS, BRAF, etc.) (**Figure 1**). In particular, tyrosine (not serine/threonine) phosphorylation of β-catenin (and loss of its degradation), leads to its nuclear translocation and oncogene transactivation (Piedra et al., 2001). Consequently, a number of Wnt inhibitors have now been developed, including those targeting tankarases 1 and 2 (affecting AXIN2 degradation), porcupine, and disheveled (Voronkov and Krauss, 2013). Thus, while inhibitors of canonical Wnt signaling may well be beneficial, even more ideal therapeutics might target the downstream effectors that act as convergence points for several pathways (e.g., TCF/LEF, TWIST, MYC, etc.) (Voronkov and Krauss, 2013).

### Epigenome-Modulating Agents

In addition to Wnt inhibitors, we posit that epigenetic transcriptional derepressors might be a complimentary means of enhancing the efficacy of embryonic pathway-targeting therapies. For example, DNA methylation specifically silences a number of tumor suppressor genes (e.g., *miR-34*, *miR-200* family, *SFRP*, etc.), while global epigenomic alterations underlie the phenotypic plasticity of EMT/stemness. Such dedifferentiating phenotypic alterations correlate with widespread trimethylation of histone H3, lysine 27 (H3K27me3), a transcriptionally repressive modification catalyzed by the Polycomb group protein, and histone methyltransferase, EZH2 (Widschwendter et al., 2007; Friedman et al., 2009; Yan and Guo, 2015) (**Figure 1**). Most recently, the retinoblastoma-binding protein-2, RBP2 (JARED1A, KDM5a), a histone H3 lysine 4 (H3K4, an activating modification) demethylase, was discovered in various other cancers as complexed with EZH2 (Pasini et al., 2008; Schuettengruber and Cavalli, 2009), and is now the subject of intense investigation for inhibitors (Zeng et al., 2010; Lin et al., 2011).

Interestingly, one study in YB5 CRC cells comprised of a screen of FDA-approved drugs that could synergize with the DNA methylation inhibitor decitabine to derepress a silenced reporter gene (Raynal et al., 2017). This screen revealed, in particular, that the antiarrhythmic proscillaridin, paired with decitabine, effected widespread epigenomic reprogramming, including silencing of two CRC oncogenes, *SYMD3* and *KDM8* (Raynal et al., 2017).

Several other recent studies support our hypothesis. In one study of chemoresistant CRC and other cell lines, histone deacetylase inhibitors (HDACIs) reversed EMT and cancer stem cell phenotypes (Wu et al., 2017), while one specific HDACI, resminostat, has now completed a Phase I clinical trial (Clinicaltrials.gov, NCT01277406) for patients with advanced CRC (Ahmed et al., 2017). Another Phase II trial of the HDACI entinostat, combined with the demethylating agent azacitidine proved tolerable, but unfortunately, poorly efficacious (Azad et al., 2017). Other HDACIs, however, in Phase I or II trials, include CUDC-907 (Curis, Inc., Lexington, MA, USA) and CXD101 (Celleron Therapeutics, Oxford, UK). Thus, while HDACIs have yet only shown efficacy against specific lymphomas, it is likely that well-designed clinical trials will validate their promise against solid tumors (particularly as adjuvant therapies).

## Summary

Much progress has been made in the treatment of CRC, including a tripling of the 5-year survival for stage IV disease, from 11 to 30 months. Nonetheless, following most first-line therapies, secondary approaches remain only minimally life-extending. Due to the essential and complex involvement of epigenetics, in CRC and most cancers, approaches to reverse the various DNA and histone modifications related to self-renewal of cancer stem cells, hold promise for more effective treatment of CRC and other malignant diseases.

## Author Contributions

CB and AT: idea generation, literature review. AT: supervised the outline, designed the figure in collaboration with CB and JR. CB, JR, and AT contributed to the writing and proofing of the manuscript. All the authors read and approved the final version of this manuscript.

## Conflict of Interest Statement

The authors declare that the research was conducted in the absence of any commercial or financial relationships that could be construed as a potential conflict of interest. The reviewer KM and handling Editor declared their shared affiliation, and the handling Editor states that the process nevertheless met the standards of a fair and objective review.
